# On the Classical Capacity of General Quantum Gaussian Measurement

**DOI:** 10.3390/e23030377

**Published:** 2021-03-21

**Authors:** Alexander Holevo

**Affiliations:** Steklov Mathematical Institute, RAS, 119991 Moscow, Russia; holevo@mi-ras.ru

**Keywords:** Gaussian measurement channel, classical capacity, Gaussian ensemble, accessible information, Gaussian maximizer

## Abstract

In this paper, we consider the classical capacity problem for Gaussian measurement channels. We establish Gaussianity of the average state of the optimal ensemble in the general case and discuss the Hypothesis of Gaussian Maximizers concerning the structure of the ensemble. Then, we consider the case of one mode in detail, including the dual problem of accessible information of a Gaussian ensemble. Our findings are relevant to practical situations in quantum communications where the receiver is Gaussian (say, a general-dyne detection) and concatenation of the Gaussian channel and the receiver can be considered as one Gaussian measurement channel. Our efforts in this and preceding papers are then aimed at establishing full Gaussianity of the optimal ensemble (usually taken as an assumption) in such schemes.

## 1. Introduction

From the viewpoint of information theory, measurements are hybrid communication channels that transform input quantum states into classical output data. As such, they are described by the classical information capacity which is the most fundamental quantity characterizing their ultimate information-processing performance [1,2,3,4]. Channels with continuous output, such as bosonic Gaussian measurements, do not admit direct embedding into properly quantum channels and, hence, require separate treatment. In particular, their output entropy is the Shannon differential entropy, instead of the quantum entropy, which completely changes the pattern of the capacity formulas. The classical capacity of multimode Gaussian measurement channels was computed in Reference [5] under so-called threshold condition (which includes phase-insensitive or gauge covariant channels as a special case [6]). The essence of this condition is that it reduces the classical capacity problem to the minimum output differential entropy problem solved in Reference [7] (in the context of quantum Gaussian channels, a similar condition was introduced and studied in References [8,9]; also see references therein).

In this paper, we approach the classical capacity problem for Gaussian measurement channels without imposing any kind of threshold condition. In particular, in the framework of quantum communication, this means that both (noisy) heterodyne and (noisy/noiseless) homodyne measurements [10,11] are treated from a common viewpoint. We prove Gaussianity of the average state of the optimal ensemble in general and discuss the Hypothesis of Gaussian Maximizers (HGM) concerning the structure of the ensemble. The proof uses the approach of the paper of Wolf, Giedke, and Cirac [12] applied to the convex closure of the output differential entropy. Then, we discuss the case of one mode in detail, including the dual problem of accessible information of a Gaussian ensemble.

In quantum communications, there are several studies of the classical capacity in the transmission scheme where not only the Gaussian channel but also the receiver is fixed, and the optimization is performed over certain set of the input ensembles (see References [10,13,14,15] and references therein). These studies are practically important in view of greater complexity of the optimal receiver in the Quantum Channel Coding (HSW) theorem (see, e.g., Reference [16]). Our findings are relevant to such a situation where the receiver is Gaussian and concatenation of the channel and the receiver can be considered as one Gaussian measurement channel. Our efforts in this and preceding papers are then aimed at establishing full Gaussianity of the optimal ensemble (usually taken as a key assumption) in such schemes.

## 2. The Measurement Channel and Its Classical Capacity

An *ensemble* $E=\left\{\pi (dx),\rho (x)\right\}$ consists of probability measure π(dx) on a standard measurable space X and a measurable family of density operators (quantum states) x→ρ(x) on the Hilbert space H of the quantum system. The *average state* of the ensemble is the barycenter of this measure:

$${\bar{\rho }}_{E}=\int_{X}\rho \left(x\right)\;\pi \left(dx\right),$$

the integral existing in the strong sense in the Banach space of trace-class operators on H.

Let M={M(dy)} be an observable (POVM) on H with the outcome standard measurable space Y. There exists a σ−finite measure μ(dy) such that, for any density operator ρ, the probability measure TrρM(dy) is absolutely continuous w.r.t. μ(dy), thus having the probability density $p_{\rho }\left(y\right)$ (one can take $\mu \left(dy\right)=\mathrm{Tr}\rho_{0}M\left(dy\right)$, where $\rho_{0}$ is a nondegenerate density operator). The affine map $M:\rho \to p_{\rho }\left(\cdot \right)$ will be called the *measurement channel*.

The joint probability distribution of x,y on X×Y is uniquely defined by the relation

$$P\left(A\times B\right)=\int_{A}\pi \left(dx\right)\mathrm{Tr}\;\rho \left(x\right)M\left(B\right)=\int_{A}\int_{B}\;p_{\rho (x)}\left(y\right)\;\pi \left(dx\right)\mu \left(dy\right),$$

where *A* is an arbitrary Borel subset of X, and *B* is that of Y. The classical Shannon information between x,y is equal to

$$I\left(E,M\right)=\int \int \pi \left(dx\right)\mu \left(dy\right)p_{\rho (x)}\left(y\right)\log\frac{p_{\rho (x)}\left(y\right)}{p_{{\bar{\rho }}_{E}}\left(y\right)}.$$

In what follows, we will consider POVMs having (uniformly) bounded operator density, M(dy)=m(y)μ(dy), with $∥m(y)∥\leq b,$ so that the probability densities $p_{\rho }\left(y\right)=\mathrm{Tr}\;\rho m\left(y\right)$ are uniformly bounded, $0\leq p_{\rho }\left(y\right)\leq b$. (The probability densities corresponding to Gaussian observables we will be dealing with possess this property). Moreover, without loss of generality [6] we can assume b=1. Then, the output differential entropy

(1) $$h_{M}\left(\rho \right)=-\int p_{\rho }\left(y\right)\log\;p_{\rho }\left(y\right)\mu \left(dy\right)$$

is well defined with values in [0,+∞] (see Reference [6] for the details). The output differential entropy is concave lower semicontinuous (w.r.t. trace norm) functional of a density operator ρ. The concavity follows from the fact that the function p→−plogp,p∈[0,1] is concave. Lower semicontinuity follows by an application of the Fatou-Lebesgue lemma from the fact that this function is nonnegative, continuous, and $\left|p_{\rho }\left(y\right)-p_{\sigma }\left(y\right)\right|\leq {∥\rho -\sigma ∥}_{1}.$

Next, we define the *convex closure of the output differential entropy* (1):

(2) $$e_{M}\left(\rho \right)=\underset{E:{\bar{\rho }}_{E}=\rho }{\inf}\int h_{M}\left(\rho \left(x\right)\right)\pi \left(dx\right),$$

which is the “measurement channel analog” of the convex closure of the output entropy for a quantum channel [17].

**Lemma** **1.** *The functional $e_{M}\left(\rho \right)$ is convex, lower semicontinuous and strongly superadditive:*

(3) $$e_{M_{1}⊗M_{2}}\left(\rho_{12}\right)\geq e_{M_{1}}\left(\rho_{1}\right)+e_{M_{2}}\left(\rho_{2}\right).$$

As it is well known, the property (3) along with the definition (2) imply *additivity*: if $\rho_{12}=\rho_{1}⊗\rho_{2}$ then

(4) $$e_{M_{1}⊗M_{2}}\left(\rho_{12}\right)=e_{M_{1}}\left(\rho_{1}\right)+e_{M_{2}}\left(\rho_{2}\right).$$

**Proof.**  The lower semicontinuity follows from the similar property of the output differential entropy much in the same way as in the case of quantum channels, treated in Reference [17], Proposition 4; also see Reference [18], Proposition 1.

Let us prove strong superadditivity. Let

(5) $$\rho_{12}=\int \rho_{12}\left(x\right)\pi \left(dx\right)$$

be a decomposition of a density operator $\rho_{12}$ on $H_{1}⊗H_{2}$, then

$$\begin{array}{cc} & p_{M_{1}⊗M_{2}}\left(y_{1},y_{2}|x\right)\\ = & \mathrm{Tr}\;\rho_{12}\left(x\right)\left[m_{1}\left(y_{1}\right)⊗m_{2}\left(y_{2}\right)\right]\\ = & \mathrm{Tr}\;\rho_{1}\left(x\right)\;m_{1}\left(y_{1}\right)\;\mathrm{Tr}\;\rho_{2}\left(y_{1},x\right)\;m_{2}\left(y_{2}\right)\\ = & p_{M_{1}}\left(y_{1}|x\right)\;p_{M_{2}}\left(y_{2}|y_{1},x\right), \end{array}$$

where $\;\rho_{1}\left(x\right)={\mathrm{Tr}}_{2}\;\rho_{12}\left(x\right),\rho_{2}\left(y_{1},x\right)=\frac{{\mathrm{Tr}}_{1}\;\rho_{12}\left(x\right)\left[m_{1}\left(y_{1}\right)⊗I_{2}\right]}{\mathrm{Tr}\;\rho_{12}\left(x\right)\left[m_{1}\left(y_{1}\right)⊗I_{2}\right]},$ so that

$$\mathrm{Tr}\;\rho_{12}\left(x\right)\left[m_{1}\left(y_{1}\right)⊗I_{2}\right]=\mathrm{Tr}\;\rho_{1}\left(x\right)\;m_{1}\left(y_{1}\right)=p_{M_{1}}\left(y_{1}|x\right),$$

and $\rho_{2}=\int \int \rho_{2}\left(y_{1},x\right)p_{M_{1}}\left(y_{1}|x\right)\pi \left(dx\right)\mu_{1}\left(dy_{1}\right)$ while $\rho_{1}=\int \rho_{1}\left(x\right)\pi \left(dx\right).$ It follows that:

$$\begin{array}{ccc} h(Y_{1},Y_{2}|X) & \equiv & \int h_{M_{1}⊗M_{2}}\left(\rho_{12}\left(x\right)\right)\pi \left(dx\right)\\ & = & \int h_{M_{1}}\left(\rho_{1}\left(x\right)\right)\pi \left(dx\right)\\ & + & \int \int h_{M_{2}}\left(\rho_{2}\left(y_{1},x\right)\right)p_{M_{1}}\left(y_{1}|x\right)\pi \left(dx\right)\mu_{1}\left(dy_{1}\right)\\ & = & h\left(Y_{1}|X\right)+h\left(Y_{2}|Y_{1},X\right), \end{array}$$

and, whence taking the infimum over decompositions (5), we obtain (3). □

Let *H* be a Hamiltonian in the Hilbert space H of the quantum system, *E* a positive number. Then, the *energy-constrained classical capacity* of the channel *M* is equal to

(6) $$C\left(M,H,E\right)=\underset{E:\mathrm{Tr}{\bar{\rho }}_{E}H\leq E}{\sup}I\left(E,M\right),$$

where maximization is over the input ensembles of states E satisfying the energy constraint $\mathrm{Tr}{\bar{\rho }}_{E}H\leq E$, as shown in Reference [5], proposition 1.

If $h_{M}\left({\bar{\rho }}_{E}\right)<+\infty$, then

(7) $$I\left(E,M\right)=h_{M}\left({\bar{\rho }}_{E}\right)-\int h_{M}\left(\rho \left(x\right)\right)\pi \left(dx\right).$$

Note that the measurement channel is entanglement-breaking [16]; hence, its classical capacity is additive and is given by the one-shot expression (6). By using (7), (2), we obtain

(8) $$C\left(M,H,E\right)=\underset{\rho :\mathrm{Tr}\rho H\leq E}{\sup}\left[h_{M}\left(\rho \right)-e_{M}\left(\rho \right)\right].$$

## 3. Gaussian Maximizers for Multimode Bosonic Gaussian Observable

Consider now multimode bosonic Gaussian system with the quadratic Hamiltonian $H=R\epsilon R^{t},$ where ϵ>0 is the energy matrix, and $R=\left[q_{1},p_{1},\ldots ,q_{s},p_{s}\right]$ is the row vector of the bosonic position-momentum observables, satisfying the canonical commutation relation

$$\left[R^{t},R\right]=i\Delta I,\;\Delta =\mathrm{diag}{\left[\begin{array}{cc} 0 & 1\\ -1 & 0 \end{array}\right]}_{\bar{1,\ldots ,s}},$$

(see, e.g., References [11,16]). This describes quantization of a linear classical system with *s* degrees of freedom, such as finite number of physically relevant electromagnetic modes on the receiver’s aperture in quantum optics.

From now on, we will consider only states with finite second moments. By S(α), we denote the set of all states ρ with the fixed correlation matrix

α=ReTrRtρR.

For *centered* states (i.e., states with vanishing first moments), the covariance matrix and the matrix of second moments coincide. We denote by $\rho_{\alpha }$ centered Gaussian state with the correlation matrix α≥±i/2Δ. For states ρ∈S(α), we have $h_{M}\left(\rho \right)\leq h_{M}\left(\rho_{\alpha }\right)<+\infty ,$ by the maximum entropy principle.

The energy constraint reduces to

(9) Spαϵ≤E.

(We denote Sp trace of s×s-matrices as distinct from trace of operators on H.)

For a fixed correlation matrix α, we will study the α-*constrained* capacity

(10) $$C\left(M;\alpha \right)=\underset{E:{\bar{\rho }}_{E}\in S\left(\alpha \right)}{\sup}I\left(E,M\right)=\underset{\rho \in S(\alpha )}{\sup}\left[h_{M}\left(\rho \right)-e_{M}\left(\rho \right)\right].$$

With the Hamiltonian $H=R\epsilon R^{t},$ the *energy-constrained classical capacity* of observable *M* is

$$C\left(M;H,E\right)=\underset{\alpha :\mathrm{Sp}\;\alpha \epsilon \leq E}{\sup}C\left(M;\alpha \right).$$

We will be interested in the approximate position-momentum measurement (observable, POVM)

(11) $$M\left(d^{2s}z\right)=D\left(z\right)\rho_{\beta }D{\left(z\right)}^{*}\frac{d^{2s}z}{{\left(2\pi \right)}^{s}}$$

where $\rho_{\beta }$ is centered Gaussian density operator with the covariance matrix β and

$$D\left(z\right)=\exp i\sum_{j=1}^{s}\left(y_{j}q_{j}-x_{j}p_{j}\right),\;z={\left[\begin{array}{ccc} x_{1},y_{1}, & \ldots , & x_{s},y_{s} \end{array}\right]}^{t}\in R^{2s}$$

are the unitary displacement operators. Thus, $\mu \left(dz\right)=\frac{d^{2s}z}{{\left(2\pi \right)}^{s}}$ and the operator-valued density of POVM (11) is $m\left(z\right)=D\left(z\right)\rho_{\beta }D{\left(z\right)}^{*}.$ In quantum optics, some authors [11,19] call such measurements (noisy) general-dyne detections.

In what follows, we will consider *n* independent copies of our bosonic system on the Hilbert space $H^{⊗n}.$ We will supply all the quantities related to k-th copy (k=1,…,n) with upper index ${}^{\left(k\right)}$, and we will use tilde to denote quantities related to the whole collection on *n* copies. Thus,

$$\tilde{z}=\left[\begin{array}{c} z^{\left(1\right)}\\ \cdots \\ z^{\left(n\right)} \end{array}\right],\;D\left(\tilde{z}\right)=D\left(z^{\left(1\right)}\right)⊗\cdots ⊗D\left(z^{\left(n\right)}\right)$$

and

$$M^{⊗n}\left(d\tilde{z}\right)=\tilde{m}\left(\tilde{z}\right)\tilde{\mu }\left(d\tilde{z}\right)=\left[m\left(z^{\left(1\right)}\right)⊗\cdots ⊗m\left(z^{\left(n\right)}\right)\right]\;\mu \left(dz^{\left(1\right)}\right)\ldots \mu \left(dz^{\left(n\right)}\right).$$

**Lemma** **2.** *Let $O={\left[O_{kl}\right]}_{k,l=1,\ldots ,n}$ be a real orthogonal n×n—matrix and U—the unitary operator on $H^{⊗n}$ implementing the linear symplectic transformation*

$$\tilde{R}=\left[\begin{array}{ccc} R^{\left(1\right)}, & \ldots , & R^{\left(n\right)} \end{array}\right]\to \tilde{R}\;O,\;$$

*so that*

(12) $$U^{*}D\left(\tilde{z}\right)U=D\left(O\;\tilde{z}\right).$$

*Then, for any state $\tilde{\rho }$ on $H^{⊗n}$,*

(13) $$e_{M^{⊗n}}\left(\tilde{\rho }\right)=e_{M^{⊗n}}\left(U\tilde{\rho }U^{*}\right).$$

**Proof.**  The covariance matrix $\tilde{\beta }$ of $\rho_{\beta }^{⊗n}$ is block-diagonal, $\tilde{\beta }={\left[\delta_{kl}\beta \right]}_{k,l=1,\ldots ,n}$; hence, $O^{t}\tilde{\beta }O=\tilde{\beta }$. Thus, we have $U^{*}\rho_{\beta }^{⊗n}U=\rho_{\beta }^{⊗n},$ and taking into account (12),

$$U^{*}\tilde{m}\left(\tilde{z}\right)U=D\left(O\;\tilde{z}\right)\rho_{\beta }^{⊗n}D{\left(O\;\tilde{z}\right)}^{*}=\tilde{m}\left(O\tilde{z}\right).$$

Therefore, for any state $\tilde{\sigma }$ on $H^{⊗n}$, the output probability density of the measurement channel $\tilde{M}=M^{⊗n}$ corresponding to the input state $U\tilde{\sigma }U^{*}$ is

(14) $$p_{U\tilde{\sigma }U^{*}}\left(\tilde{z}\right)=\mathrm{Tr}\;\left(U\tilde{\sigma }U^{*}\right)\tilde{m}\left(\tilde{z}\right)=\mathrm{Tr}\;\tilde{\sigma }\tilde{m}\left(O\tilde{z}\right)=p_{\tilde{\sigma }}\left(O\tilde{z}\right).$$

Hence, by using orthogonal invariance of the Lebesgue measure,

$$h_{M^{⊗n}}\left(U\tilde{\sigma }U^{*}\right)=h_{M^{⊗n}}\left(\tilde{\sigma }\right).$$

If $\tilde{\rho }=\int_{X}\tilde{\rho }\left(x\right)\;\pi \left(dx\right),$ then $U\tilde{\rho }U^{*}=\int_{X}\left(U\tilde{\rho }\left(x\right)\;U^{*}\right)\pi \left(dx\right),$ and taking $\tilde{\sigma }=\tilde{\rho }\left(x\right)$ in the previous formula, we deduce

$$\int_{X}h_{M^{⊗n}}\left(U\tilde{\rho }\left(x\right)U^{*}\right)\pi \left(dx\right)=\int_{X}h_{M^{⊗n}}\left(\tilde{\rho }\left(x\right)\right)\pi \left(dx\right);$$

hence, (13) follows. □

**Lemma** **3.** *Let M be the Gaussian measurement (11). For any state ρ with finite second moments, $e_{M}\left(\rho \right)\geq e_{M}\left(\rho_{\alpha }\right)$, where α is the covariance matrix of ρ.*

**Proof.**  The proof follows the pattern of Lemma 1 from the paper of Wolf, Giedke, and Cirac [12]. Without loss of generality, we can assume that ρ is centered. We have

(15) $$e_{M}\left(\rho \right)\overset{\left(1\right)}{=}\frac{1}{n}e_{M^{⊗n}}\left(\rho^{⊗n}\right)\overset{\left(2\right)}{=}\frac{1}{n}e_{M^{⊗n}}\left(\tilde{\rho }\right)\overset{\left(3\right)}{\geq }\frac{1}{n}\sum_{k=1}^{n}e_{M}\left({\tilde{\rho }}^{\left(k\right)}\right),$$

where $\tilde{\rho }=U\rho^{⊗n}U^{*}$ with symplectic unitary *U* in $H^{⊗n},\;$ corresponding to an orthogonal matrix *O* as in Lemma 2, and ${\tilde{\rho }}^{\left(k\right)}$ is the k-th partial state of $\tilde{\rho }.$

Step (1) follows from the additivity (4). Step (2) follows from Lemma 2, and step (3) follows from the superadditivity of $e_{M}$ (Lemma 1). The final step of the proof,

(16) $$\underset{n\to \infty }{lim inf}\frac{1}{n}\sum_{k=1}^{n}e_{M}\left({\tilde{\rho }}^{\left(k\right)}\right)\geq e_{M}\left(\rho_{\alpha }\right),$$

uses ingeniously constructed *U* from Reference [12] and lower semicontinuity of $e_{M}$ (Lemma 1). Namely, $n=2^{m},$ and *U* corresponds via (12) to the following special orthogonal matrix

$$O={\left[O_{kl}\right]}_{k,l=1,\ldots ,n}=H^{⊗m},\;H=\frac{1}{\sqrt{2}}\left[\begin{array}{cc} 1 & 1\\ 1 & -1 \end{array}\right].$$

Every row of the n×n−matrix *O*, except the first one which has all the elements 1, has $n/2=2^{m-1}$ elements equal to 1 and n/2 elements equal to −1. Then, the quantum characteristic function of the states ${\tilde{\rho }}^{\left(k\right)},k=2,\ldots ,n$ is equal to $\phi {\left(z/\sqrt{n}\right)}^{n/2}\phi {\left(-z/\sqrt{n}\right)}^{n/2}$, where ϕ(z) is the quantum characteristic function of the state ρ. This allows to apply Quantum Central Limit Theorem [20] to show that ${\tilde{\rho }}^{\left(k\right)}\to \rho_{\alpha }$ as n→∞, in a uniform way, implying (16); see Reference [12] for details. □

**Theorem** **1.** *The optimizing density operator ρ in (10) is the (centered) Gaussian density operator $\rho_{\alpha }:$*

(17) $$C\left(M;\alpha \right)=h_{M}\left(\rho_{\alpha }\right)-e_{M}\left(\rho_{\alpha }\right),$$

*and, hence,*

(18) $$C\left(M,H,E\right)=\max_{\alpha :\mathrm{Sp}\;\alpha \;\epsilon \;\leq E}C\left(M;\alpha \right)=\max_{\alpha :\mathrm{Sp}\;\alpha \;\epsilon \;\leq E}\left[h_{M}\left(\rho_{\alpha }\right)-e_{M}\left(\rho_{\alpha }\right)\right].$$

**Proof.**  Lemma 3 implies that, for any ρ with finite second moments, $e_{M}\left(\rho \right)\geq e_{M}\left(\rho_{\alpha }\right)$, where α is the covariance matrix of ρ. On the other hand, by the maximum entropy principle, $h_{M}\left(\rho \right)\leq h_{M}\left(\rho_{\alpha }\right)$. Hence, (17) is maximized by a Gaussian density operator. □

**Remark** **1.** *The proof of Lemma 2 and, hence, of Theorem 1 can be extended to a general Gaussian observable M in the sense of References [16,21], defined via operator-valued characteristic function of the form*

(19) $$\phi_{M}\left(w\right)=\exp\left(i\;R\;Kw-\frac{1}{2}w^{t}\gamma w\right),$$

*where K is a scaling matrix, γ is the measurement noise covariance matrix, and $\gamma \geq \pm \frac{i}{2}K^{t}\Delta K$. Then, the Fourier transform of the measurement probability density $p_{\rho }\left(z\right)$ is equal to $\mathrm{Tr}\;\rho \phi_{M}\left(w\right)$, and one can use this function to obtain generalization of the relation (14) for the measurement probability densities. The case (11) corresponds to the type 1 Gaussian observable [21] with $K=I_{2s},\gamma =\beta$. However, (19) also includes type 2 and 3 observables (noisy and noiseless multimode homodyning), in which case K is a projection onto an isotropic subspace of Z (i.e., one on which the symplectic form* Δ *vanish.)*

**Remark** **2.** *Theorem 1 establishes Gaussianity of the average state of the optimal ensemble for a general Gaussian measurement channel. However, Gaussian average state can appear in a non-Gaussian ensemble. An immediate example is thermal state represented as a mixture of the Fock states with geometric distribution. Thus, Theorem 1 does not necessarily imply full Gaussianity of the optimal ensemble as formulated in the following conjecture.*

**Hypothesis of Gaussian Maximizers** **(HGM).** *Let M be an arbitrary Gaussian measurement channel. Then, there exists an optimal Gaussian ensemble for the convex closure of the output differential entropy (2) with Gaussian ρ and, hence, for the energy-constrained classical capacity (6) of the channel M. More explicitly, the ensemble consists of (properly squeezed) coherent states with the displacement parameter having Gaussian probability distribution.*

For Gaussian measurement channels of the type 1 (essentially of the form (11), see Reference [21] for complete classification) and Gaussian states $\rho_{\alpha }$ satisfying the “threshold condition”, we have

(20) $$e_{M}\left(\rho_{\alpha }\right)=\min_{\rho }h_{M}\left(\rho \right),$$

with the minimum attained on a squeezed coherent state, which implies the validity of the HGM and an efficient computation of C(M,H,E); see Reference [5]. On the other hand, the problem remains open in the case where the “threshold condition” is violated, and in particular, for all Gaussian measurement channels of the type 2 (noisy homodyning), with the generic example of the energy-constrained approximate measurement of the position $\left[q_{1},\ldots ,q_{s}\right]$ subject to Gaussian noise (see Reference [22], where the entanglement-assisted capacity of such a measurement was computed). In the following section, we will touch upon the HGM in this case for one mode system.

## 4. Gaussian Measurements in One Mode

Our framework in this section will be one bosonic mode described by the canonical position and momentum operators q,p. We recall that

$$D\left(x,y\right)=\exp i\left(yq-xp\right),\;x,y\in R$$

are the unitary displacement operators.

We will be interested in the observable

(21) $$M\left(dxdy\right)=D\left(x,y\right)\rho_{\beta }D{\left(x,y\right)}^{*}\frac{dxdy}{2\pi },$$

where $\rho_{\beta }$ is centered Gaussian density operator with the covariance matrix

(22) $$\beta =\left[\begin{array}{cc} \beta_{q} & 0\\ 0 & \beta_{p} \end{array}\right];\;\beta_{q}\beta_{p}\geq \frac{1}{4}.$$

Let $\rho_{\alpha }$ be a centered Gaussian density operator with the covariance matrix

(23) $$\alpha =\left[\begin{array}{cc} \alpha_{q} & 0\\ 0 & \alpha_{p} \end{array}\right].$$

The problem is, to compute $e_{M}\left(\rho_{\alpha }\right)$ and, hence, the classical capacity C(M,H,E) for the oscillator Hamiltonian $H=\frac{1}{2}\left(q^{2}+p^{2}\right)$ (as shown in the Appendix of Reference [22], we can restrict to Gaussian states $\rho_{\alpha }$ with the diagonal covariance matrix in this case). The energy constraint (9) takes the form

(24) $$\alpha_{q}+\alpha_{p}\leq 2E.$$

The measurement channel corresponding to POVM (21) acts on the centered Gaussian state $\rho_{\alpha }$ by the formula

(25) $$\begin{array}{ccc} M & : & \rho_{\alpha }\to p_{\rho_{\alpha }}\left(x,y\right)\\ & = & \frac{1}{\sqrt{2\pi \left(\alpha_{q}+\beta_{q}\right)\left(\alpha_{p}+\beta_{p}\right)}}\exp\left[-\frac{x^{2}}{2\left(\alpha_{q}+\beta_{q}\right)}-\frac{y^{2}}{2\left(\alpha_{p}+\beta_{p}\right)}\right], \end{array}$$

so that

(26) $$h_{M}\left(\rho_{\alpha }\right)=\frac{1}{2}\log\left(\alpha_{q}+\beta_{q}\right)\left(\alpha_{p}+\beta_{p}\right)+c.$$

In this expression, *c* is a fixed constant depending on the normalization of the underlying measure μ in (1). It does not enter the information quantities which are differences of the two differential entropies.

Assuming validity of the HGM, we will optimize over ensembles of squeezed coherent states

$$\rho_{x,y}=D\left(x,y\right)\;\rho_{\Lambda }D{\left(x,y\right)}^{*},\;\left(x,y\right)\in R^{2},$$

where $\rho_{\Lambda }$ is centered Gaussian state with correlation matrix $\Lambda =\left[\begin{array}{cc} \delta & 0\\ 0 & 1/\left(4\delta \right) \end{array}\right],$ and the vector (x,y) has centered Gaussian distribution with covariance matrix $\left[\begin{array}{cc} \gamma_{q} & 0\\ 0 & \gamma_{p} \end{array}\right].$ Then, the average state ${\bar{\rho }}_{E}$ of the ensemble is centered Gaussian $\rho_{\alpha }$ with the covariance matrix (23), where

$$\alpha_{q}=\gamma_{q}+\delta ,\;\alpha_{p}=\gamma_{p}+1/\left(4\delta \right);$$

hence,

(27) $$\frac{1}{4\alpha_{p}}\leq \delta \leq \alpha_{q}.$$

For this ensemble,

$$\int h_{M}\left(\rho_{x,y}\right)\pi \left(dx\;dy\right)=h_{M}\left(\rho_{\Lambda }\right)=\frac{1}{2}\log\left(\delta +\beta_{q}\right)\left(1/\left(4\delta \right)+\beta_{p}\right)+c.$$

Then, the hypothetical value:

(28) $$e_{M}\left(\rho_{\alpha }\right)=\min_{1/\left(4\alpha_{p}\right)\leq \delta \leq \alpha_{q}}\frac{1}{2}\log\left(\delta +\beta_{q}\right)\left(1/\left(4\delta \right)+\beta_{p}\right)+c.$$

The derivative of the minimized expression vanishes for $\delta =\frac{1}{2}\sqrt{\frac{\beta_{q}}{\beta_{p}}}.$ Thus, depending on the position of this value with respect to the interval (27), we obtain three possibilities):

Here, the column C corresponds to the case where the “threshold condition” holds, implying (20). Then the full validity of the HGM in much more general multimode situation was established in Reference [5]. All the quantities in this column, as well as the value of C(M,H,E) in the central column of Table 2, were obtained in that paper as an example. On the other hand, the HGM remains open in the cases of mutually symmetric columns L and R (for the derivation of the quantities in column L of Table 1 and Table 2 see Appendix A).

Maximizing C(M;α) over $\alpha_{q},\alpha_{p}$ which satisfy the energy constraint (24) (with the equality): $\alpha_{q}+\alpha_{p}=2E$, we obtain C(M,H,E) depending on the signal energy *E* and the measurement noise variances $\beta_{q},\beta_{p}:$

**Table 2:** The values of the capacity C(M,H,E).

L: HGM open	C: HGM valid [5]	R: HGM open
$\beta_{q}\leq \beta_{p};E<E\left(\beta_{p},\beta_{q}\right)$	$E\geq E\left(\beta_{p},\beta_{q}\right)\vee E\left(\beta_{q},\beta_{p}\right)$	$\beta_{p}\leq \beta_{q};E<E\left(\beta_{q},\beta_{p}\right)$
$\log\left(\frac{\sqrt{1+8E\beta_{q}+4\beta_{q}^{2}}-1}{2\beta_{q}}\right)$	$\log\left(\frac{E+\left(\beta_{q}+\beta_{p}\right)/2}{\sqrt{\beta_{q}\beta_{p}}+1/2}\right)$	$\log\left(\frac{\sqrt{1+8E\beta_{p}+4\beta_{p}^{2}}-1}{2\beta_{p}}\right)$

where we introduced the “energy threshold function”

$$E\left(\beta_{1},\beta_{2}\right)=\frac{1}{2}\left(\beta_{1}-\beta_{2}+\sqrt{\frac{\beta_{1}}{\beta_{2}}}\right).$$

In the gauge invariant case when $\beta_{q}=\beta_{p}=\beta$, the threshold condition amounts to E≥1/2, which is fulfilled by definition, and the capacity formula gives the expression $\log\left(\frac{E+\beta }{\beta +1/2}\right)$ equivalent to one obtained in Hall’s 1994 paper [13].

Let us stress that, opposite to column C, the values of C(M,H,E) in the L and R columns are hypothetic, conditional upon validity of the HGM. Looking into the left column, one can see that C(M;α) and C(M,H,E) do not depend at all on $\beta_{p}.$ Thus, we can let the variance of the momentum *p* measurement noise $\beta_{p}\to +\infty ,$ and, in fact, set $\beta_{p}=+\infty ,$ which is equivalent to the approximate measurement only of the position *q* described by POVM

(29) $$M\left(dx\right)=\exp\left[-\frac{{\left(q-x\right)}^{2}}{2\beta_{q}}\right]\frac{dx}{\sqrt{2\pi \beta_{q}}}=D\left(x,0\right)e^{-q^{2}/2\beta_{q}}D{\left(x,0\right)}^{*}\frac{dx}{\sqrt{2\pi \beta_{q}}},$$

which belongs to type 2 according to the classification of Reference [21]. In other words, one makes the “classical” measurement of the observable

$$X=q+\xi ,\;\xi ∼N(0,\beta_{q}),$$

with the quantum energy constraint $\mathrm{Tr}\;\rho (q^{2}+p^{2})\leq 2E$.

The measurement channel corresponding to POVM (29) acts on the centered Gaussian state $\rho_{\alpha }\;$ by the formula

(30) $$M:\rho_{\alpha }\to p_{\rho_{\alpha }}\left(x\right)=\frac{1}{\sqrt{2\pi \left(\alpha_{q}+\beta_{q}\right)}}\exp\left[-\frac{x^{2}}{2\left(\alpha_{q}+\beta_{q}\right)}\right].$$

In this case, we have

(31) $$\begin{array}{ccc} h_{M}\left(\rho_{\alpha }\right) & = & \frac{1}{2}\log\left(\alpha_{q}+\beta_{q}\right)+c, \end{array}$$

(32) $$\begin{array}{ccc} e_{M}\left(\rho_{\alpha }\right) & = & \frac{1}{2}\log\left(1/\left(4\alpha_{p}\right)+\beta_{q}\right)+c, \end{array}$$

which differ from the values in the case of finite $\beta_{p}\to +\infty$ by the absence of the factor $\left(\alpha_{p}+\beta_{p}\right)$ under the logarithms, while the difference $C\left(M;\alpha \right)=h_{M}\left(\rho_{\alpha }\right)-e_{M}\left(\rho_{\alpha }\right)$ and the capacity C(M,H,E) have the same expressions as in that case (column L).

For $\beta_{q}=0$ (sharp position measurement, type 3 of Reference [21]), the HGM is valid with

C(M,H,E)=log2E.

**Figure 1:**
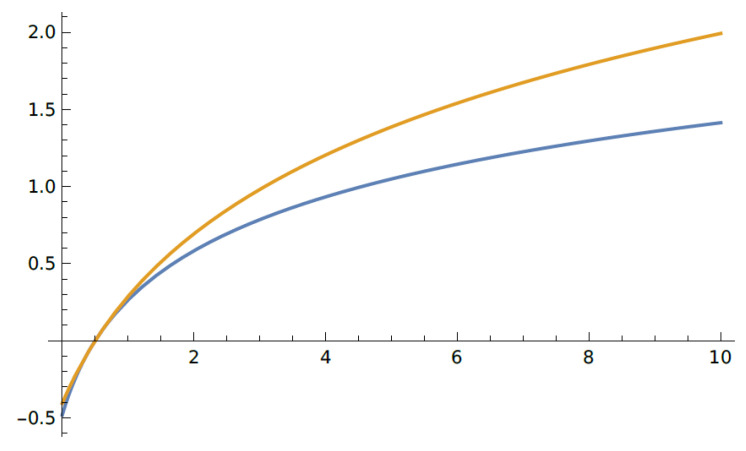
(color online) The Gaussian classical capacity (A6) and the upper bound (33) (β=1).

This follows from the general upper bound (Figure 1)

(33) $$C\left(M,H,E\right)\leq \log\left(1+\frac{E-1/2}{\beta_{q}+1/2}\right)=\log\left(\frac{2(E+\beta_{q})}{1+2\beta_{q}}\right)$$

for $\beta_{q}\geq 0$ (Equation (28) in Reference [23]; also see Equation (5.39) in Reference [10]).

## 5. The Dual Problem: Accessible Information

Let us sketch here *ensemble-observable duality* [1,2,4] (see Reference [6] for details of mathematically rigorous description in the infinite dimensional case).

Let $E=\left\{\pi (dx),\rho (x)\right\}$ be an ensemble, μ(dy) a σ−finite measure and $M=\left\{M(dy)\right\}$ an observable having operator density m(y)=M(dy)/μ(dy) with values in the algebra of bounded operators in H. The dual pair ensemble-observable $\left\{E^{\prime },M^{\prime }\right\}$ is defined by the relations

(34) $$E^{\prime }:\;\pi^{\prime }\left(dy\right)=\mathrm{Tr}{\bar{\rho }}_{E}\;M\left(dy\right),\;\rho^{\prime }\left(y\right)=\frac{{\bar{\rho }}_{E}^{1/2}m\left(y\right){\bar{\rho }}_{E}^{1/2}}{\mathrm{Tr}{\bar{\rho }}_{E}\;m\left(y\right)};$$

(35) $$M^{\prime }:\;M^{\prime }\left(dx\right)={\bar{\rho }}_{E}^{-1/2}\rho \left(x\right){\bar{\rho }}_{E}^{-1/2}\pi \left(dx\right).$$

Then, the average states of both ensembles coincide

(36) $${\bar{\rho }}_{E}={\bar{\rho }}_{E^{\prime }}$$

and the joint distribution of x,y is the same for both pairs (E,M) and $\left(E^{\prime },M^{\prime }\right)$ so that

(37) $$I\left(E,M\right)=I\left(E^{\prime },M^{\prime }\right).$$

Moreover,

(38) $$\underset{M}{\sup}I\left(E,M\right)=\underset{E^{\prime }:{\bar{\rho }}_{E^{\prime }}={\bar{\rho }}_{E}}{\sup}I\left(E^{\prime },M^{\prime }\right),$$

where the supremum in the right-hand side is taken over all ensembles $E^{\prime }$ satisfying the condition ${\bar{\rho }}_{E^{\prime }}={\bar{\rho }}_{E}$. It can be shown (Reference [6], Proposition 4), that the supremum in the lefthand side remains the same if it is taken over *all* observables *M* (not only of the special kind with the density we started with), and then it is called the *accessible information* A(E) of the ensemble E. Thus,

$$A\left(E\right)=\underset{E^{\prime }:{\bar{\rho }}_{E^{\prime }}={\bar{\rho }}_{E}}{\sup}I\left(E^{\prime },M^{\prime }\right).$$

Since the application of the duality to the pair $\left\{E^{\prime },M^{\prime }\right\}$ results in the initial pair $\left\{E,M\right\},$ we also have

$$A\left(E^{\prime }\right)=\underset{M^{\prime }}{\sup}I\left(E^{\prime },M^{\prime }\right)=\underset{E:{\bar{\rho }}_{E}={\bar{\rho }}_{E^{\prime }}}{\sup}I\left(E,M\right).$$

Coming to the case of bosonic mode, we fix the Gaussian state $\rho_{\alpha }$ and restrict to ensembles E with ${\bar{\rho }}_{E}=\rho_{\alpha }.$ Let *M* be the measurement channel corresponding to POVM (21). Then, according to formulas (34), the dual ensemble $E^{\prime }=\left\{p^{\prime }\left(x,y\right),\;\rho^{\prime }\left(x,y\right)\right\},$ where $p^{\prime }\left(x,y\right)$ is the Gaussian probability density (25) and

$$\;\rho^{\prime }\left(x,y\right)={\left[p^{\prime }\left(x,y\right)\right]}^{-1}\sqrt{\rho_{\alpha }}D\left(x,y\right)\rho_{\beta }D{\left(x,y\right)}^{*}\sqrt{\rho_{\alpha }}.$$

By using the formula for $\sqrt{\rho_{1}}\rho_{2}\sqrt{\rho_{1}}$, where $\rho_{1},\rho_{2}$ are Gaussian operators (see Reference [24] and also Corollary in the Appendix of Reference [25]), we obtain

$$\rho^{\prime }\left(x,y\right)=D\left(x^{\prime },y^{\prime }\right)\rho_{\alpha^{\prime }}D{\left(x^{\prime },y^{\prime }\right)}^{*}=\rho_{\alpha^{\prime }}\left(x^{\prime },y^{\prime }\right),$$

where

(39) $$\alpha^{\prime }=\alpha -\gamma^{\prime },\;\gamma^{\prime }=\kappa {\left(\alpha +\beta \right)}^{-1}\kappa ,\;\left[\begin{array}{c} x^{\prime }\\ y^{\prime } \end{array}\right]=\kappa {\left(\alpha +\beta \right)}^{-1}\left[\begin{array}{c} x\\ y \end{array}\right],$$

and

(40) $$\kappa =\sqrt{I+{\left(2\alpha \Delta^{-1}\right)}^{-2}}\;\alpha =\alpha \sqrt{I+{\left(2\Delta^{-1}\alpha \right)}^{-2}}.$$

Since ${\left[\begin{array}{cc} x & y \end{array}\right]}^{t}∼N\left(0,\alpha +\beta \right),$ then, from second and third equations in (39), we obtain ${\left[\begin{array}{cc} x^{\prime } & y^{\prime } \end{array}\right]}^{t}∼N\left(0,\kappa {\left(\alpha +\beta \right)}^{-1}\kappa \right)=N\left(0,\gamma^{\prime }\right).$ By denoting $p_{\gamma^{\prime }}\left(x^{\prime },y^{\prime }\right)$, the density of this normal distribution, we can equivalently rewrite the ensemble $E^{\prime }$ as $E^{\prime }=\left\{p_{\gamma^{\prime }}\left(x^{\prime },y^{\prime }\right),\;\rho_{\alpha^{\prime }}\left(x^{\prime },y^{\prime }\right)\right\}$ with the average state $\rho_{\alpha },$$\alpha =\alpha^{\prime }+\gamma^{\prime }.$ Then, HGM is equivalent to the statement

$$A\left(E^{\prime }\right)=C\left(M;\alpha \right),$$

where the values of $C\left(M;\alpha \right)$ are given in Table 1; however, they should be reexpressed in terms of the ensemble parameters $\gamma^{\prime },\alpha^{\prime }$. In Reference [25], we treated the case C in multimode situation, establishing that the optimal measurement is Gaussian, and described it. Here, we will discuss the case L (R is similar) and show that, for large $\beta_{p}$ (including $\beta_{p}=+\infty$), the HGM is equivalent to the following: the value of the accessible information

(41) $$A\left(E^{\prime }\right)=C\left(M;\alpha \right)=\frac{1}{2}\log\frac{\alpha_{q}+\beta_{q}}{\frac{1}{4\alpha_{p}}+\beta_{q}}$$

is attained on the sharp position measurement $M_{0}^{\prime }\left(d\xi \right)=|\xi \rangle \langle \xi |d\xi$ (in fact, this refers to the whole domain L: $\frac{1}{2}\sqrt{\frac{\beta_{q}}{\beta_{p}}}<\frac{1}{4\alpha_{p}},$ which, however, has rather cumbersome description in the new variables $\gamma^{\prime },\alpha^{\prime }$, cf. Reference [25]).

In the one mode case we are considering, the matrix α is given by (23), β—by (22), and $\Delta =\left[\begin{array}{cc} 0 & 1\\ -1 & 0 \end{array}\right],$ so that ${\left(2\Delta^{-1}\alpha \right)}^{2}=-\left(4\alpha_{q}\alpha_{p}\right)I.$ Computations according to (39) and (40) give

(42) $$\alpha^{\prime }=\left[\begin{array}{cc} \alpha_{q}^{\prime } & 0\\ 0 & \alpha_{p}^{\prime } \end{array}\right]=\left[\begin{array}{cc} \frac{\alpha_{q}\left(\beta_{q}+1/\left(4\alpha_{p}\right)\right)}{\alpha_{q}+\beta_{q}} & 0\\ 0 & \frac{\alpha_{p}\left(\beta_{p}+1/\left(4\alpha_{q}\right)\right)}{\alpha_{p}+\beta_{p}} \end{array}\right].$$

But under the sharp position measurement $M_{0}^{\prime }\left(d\xi \right)=|\xi \rangle \langle \xi |d\xi ,$ one has (in the formulas below, p(ξ)=N(m,α) means that p(ξ) is Gaussian probability density with mean *m* and variance α):

$$p\left(\xi |x^{\prime },y^{\prime }\right)=\langle \xi |\;\rho_{\alpha^{\prime }}\left(x^{\prime },y^{\prime }\right)|\xi \rangle =N\left(x^{\prime },\alpha_{q}^{\prime }\right),$$

while $\langle \xi |\;\rho_{\alpha }|\xi \rangle =N\left(0,\alpha_{q}\right)$ (note that ${\bar{\rho }}_{E^{\prime }}={\bar{\rho }}_{E}=\rho_{\alpha }$), and

(43) $$\begin{array}{ccc} I\left(E^{\prime },M_{0}^{\prime }\right) & = & \frac{1}{2}\left[\log\left(\alpha_{q}^{\prime }+\gamma_{q}^{\prime }\right)-\log\alpha_{q}^{\prime }\right]\\ & = & \frac{1}{2}\left[\log\alpha_{q}-\log\frac{\alpha_{q}\left(\beta_{q}+1/4\alpha_{p}\right)}{\left(\alpha_{q}+\beta_{q}\right)}\right]\\ & = & \frac{1}{2}\log\frac{\left(\alpha_{q}+\beta_{q}\right)}{\left(\beta_{q}+1/4\alpha_{p}\right)}, \end{array}$$

which is identical to the expression in (41).

In the case of the position measurement channel *M* corresponding to POVM (29) ($\beta_{p}=+\infty )$, we have $\alpha_{p}^{\prime }=\alpha_{p};$ otherwise, the argument is essentially the same. Thus, we obtain that the HGM concerning $e_{M}\left(\rho \right)$ in case L is equivalent to the following:

*The accessible information of a Gaussian ensemble $E^{\prime }=\left\{p^{\prime }\left(x\right),\;\rho^{\prime }\left(x\right)\right\},$ where*

$$p^{\prime }\left(x\right)=N\left(0,\gamma_{q}^{\prime }\right),\;\rho^{\prime }\left(x\right)=D\left(x,0\right)\rho_{\alpha^{\prime }}D{\left(x,0\right)}^{*},\;$$

*is given by the expression (43) and attained on the sharp position measurement $M_{0}^{\prime }\left(dx\right)=|\xi \rangle \langle \xi |d\xi .$*

## 6. Discussion

In this paper, we investigated the classical capacity problem for Gaussian measurement channels. We established Gaussianity of the average state of the optimal ensemble in full generality and discussed the Hypothesis of Gaussian Maximizers concerning the detailed structure of the ensemble. Gaussian systems form the backbone of information theory with continuous variables, both in the classical and in the quantum case. Starting from them, other, non-linear models can be constructed and investigated. Therefore, the quantum Gaussian models must be studied exhaustively. Despite the progress made, there are still intriguing gaps along this way. A major problem remains the proof (or refutation) of the hypothesis of Gaussian optimizers for various entropy characteristics of quantum Gaussian systems and channels. So far, the proof of this hypothesis in special cases required tricky and special constructions, such as in the path-breaking paper [7] concerning gauge-covariant channels, or in Section 3 of the present work concerning general Gaussian measurement channels. It seems plausible that quantum Gaussian systems may have some as yet undiscovered structural property, from which a proof of this hypothesis in its maximum generality would follow in a natural way.

## Figures and Tables

**Table 1:** The three parameter ranges.

range	L: $\frac{1}{2}\sqrt{\frac{\beta_{q}}{\beta_{p}}}<\frac{1}{4\alpha_{p}}$	C:$\frac{1}{4\alpha_{p}}\leq \frac{1}{2}\sqrt{\frac{\beta_{q}}{\beta_{p}}}\leq \alpha_{q}$	R: $\alpha_{q}<\frac{1}{2}\sqrt{\frac{\beta_{q}}{\beta_{p}}}$
HGM	open	valid	open
$\delta_{opt}$	$1/\left(4\alpha_{p}\right)$	$\frac{1}{2}\sqrt{\frac{\beta_{q}}{\beta_{p}}}$	$\alpha_{q}$
$e_{M}\left(\rho_{\alpha }\right)-c$	$\frac{1}{2}\log\left[\left(\frac{1}{4\alpha_{p}}+\beta_{q}\right)\right.$	$\log\left(\sqrt{\beta_{q}\beta_{p}}+1/2\right)$	$\frac{1}{2}\log\left[\left(\frac{1}{4\alpha_{q}}+\beta_{p}\right)\right.$
	$\times \left(\alpha_{p}+\beta_{p}\right)]$		$\times \left(\alpha_{q}+\beta_{q}\right)]$
C(M;α)	$\frac{1}{2}\log\frac{\alpha_{q}+\beta_{q}}{\frac{1}{4\alpha_{p}}+\beta_{q}}$	$\frac{1}{2}\log\frac{\left(\alpha_{q}+\beta_{q}\right)\left(\alpha_{p}+\beta_{p}\right)}{{\left(\sqrt{\beta_{q}\beta_{p}}+1/2\right)}^{2}}$	$\frac{1}{2}\log\frac{\alpha_{p}+\beta_{p}}{\frac{1}{4\alpha_{q}}+\beta_{p}}$

## Appendix A. Case L in Tables 1 and 2

By taking the Gaussian ensemble parameters in (28) as

(A1) $$\delta =1/\left(4\alpha_{p}\right),\;\gamma_{p}=0,\;\gamma_{q}=\alpha_{q}-1/\left(4\alpha_{p}\right),$$

we get the hypothetic value

(A2) $$e_{M}\left(\rho_{\alpha }\right)=\frac{1}{2}\log\left(\frac{1}{4\alpha_{p}}+\beta_{q}\right)\left(\alpha_{p}+\beta_{p}\right)+c,$$

hence taking into account (26),

(A3) $$C_{Gauss}\left(M;\alpha \right)=h_{M}\left(\rho_{\alpha }\right)-e_{M}\left(\rho_{\alpha }\right)=\frac{1}{2}\log\frac{\alpha_{q}+\beta_{q}}{\frac{1}{4\alpha_{p}}+\beta_{q}}.$$

The Gaussian constrained capacity is

(A4) $$\begin{array}{ccc} C_{Gauss}\left(M,H,E\right) & = & \max_{\alpha_{q}+\alpha_{q}\leq 2E}\frac{1}{2}\left[\log\left(\alpha_{q}+\beta_{q}\right)-\log\left(1/\left(4\alpha_{p}\right)+\beta_{q}\right)\right]\\ & = & \max_{\alpha_{p}}\frac{1}{2}\left[\log\left(2E-\alpha_{p}+\beta_{q}\right)-\log\left(1/\left(4\alpha_{p}\right)+\beta_{q}\right)\right], \end{array}$$

where, in the second line, we took the maximal value $\alpha_{q}=2E-\alpha_{p}$. Differentiating, we obtain the equation for the optimal value $\alpha_{p}$:

$$4\beta_{q}\alpha_{p}^{2}+2\alpha_{p}-\left(2E+\beta_{q}\right)=0,$$

the positive solution of which is

(A5) $$\alpha_{p}=\frac{1}{4\beta_{q}}\left(\sqrt{1+8E\beta_{q}+4\beta_{q}^{2}}-1\right),$$

whence

(A6) $$C_{Gauss}\left(M,H,E\right)=\log\left(\frac{\sqrt{1+8E\beta_{q}+4\beta_{q}^{2}}-1}{2\beta_{q}}\right).$$

The parameters of the optimal Gaussian ensemble are obtained by substituting the value (A5) into (A1) with $\alpha_{q}=2E-\alpha_{p}$.

The above derivation concerns the measurement (21) ($\beta_{p}<\infty ).$ The case of the measurement (29) ($\beta_{p}=+\infty )$ is treated similarly, with (A2), (26) replaced by (32), (31). Notably, in this case, the expression (A6) coincides with the one obtained in Reference [13] by optimizing the information from applying sharp position measurement to noisy optimally squeezed states (the author is indebted to M. J. W. Hall for this observation).
